# Coronary disease is not associated with robust alterations in inflammatory gene expression in human epicardial fat

**DOI:** 10.1172/jci.insight.124859

**Published:** 2019-10-17

**Authors:** Timothy P. Fitzgibbons, Nancy Lee, Khanh-Van Tran, Sara Nicoloro, Mark Kelly, Stanley K.C. Tam, Michael P. Czech

**Affiliations:** 1 Department of Medicine and; 2 Program in Molecular Medicine, University of Massachusetts (UMass) Medical School, Worcester, Massachusetts, USA.; 3 Department of Surgery, St. Elizabeth’s Medical Center, Brighton, Massachusetts, USA.

**Keywords:** Cardiology, Inflammation, Adipose tissue, Atherosclerosis, Obesity

## Abstract

Epicardial adipose tissue (EAT) is the visceral fat depot of the heart. Inflammation of EAT is thought to contribute to coronary artery disease (CAD). Therefore, we hypothesized that the EAT of patients with CAD would have increased inflammatory gene expression compared with controls without CAD. Cardiac surgery patients with (*n* = 13) or without CAD (*n* = 13) were consented, and samples of EAT and subcutaneous adipose tissue (SAT) were obtained. Transcriptomic analysis was performed using Affymetrix Human Gene 1.0 ST arrays. Differential expression was defined as a 1.5-fold change (ANOVA *P* < 0.05). Six hundred ninety-three genes were differentially expressed between SAT and EAT in controls and 805 in cases. Expression of 326 genes was different between EAT of cases and controls; expression of 14 genes was increased in cases, while 312 were increased in controls. Quantitative reverse transcription PCR confirmed that there was no difference in expression of CCL2, CCR2, TNF-α, IL-6, IL-8, and PAI1 between groups. Immunohistochemistry showed more macrophages in EAT than SAT, but there was no difference in their number or activation state between groups. In contrast to prior studies, we did not find increased inflammatory gene expression in the EAT of patients with CAD. We conclude that the specific adipose tissue depot, rather than CAD status, is responsible for the majority of differential gene expression.

## Abstract 2

In humans without atherosclerosis there is increased mRNA expression of the orphan nuclear hormone receptors in epicardial fat.

## Introduction

Obesity is a known risk factor for type 2 diabetes mellitus (T2DM). T2DM conveys increased risk for the development of cardiovascular disease (CVD). In fact, diabetes mellitus is considered a “risk equivalent” of established CVD itself (1). Obese people with visceral, rather than subcutaneous, adiposity are at greater risk for the development of T2DM and incident CVD. In the setting of chronic caloric excess, an inflammatory response is initiated in visceral adipose tissue (VAT) (2). The stimulus for this inflammatory response remains unknown. VAT inflammation results in adipocyte dysfunction, including failure to store free fatty acids as triglyceride and increased lipolysis. Excess free fatty acids are released into the circulation and deposited in ectopic tissues, such as the liver, skeletal muscle, heart, and pancreas. Ectopic lipid storage results in insulin resistance in target organs (2, 3). This paradigm has dominated our understanding of T2DM since the 1990s (4, 5). It follows that antiinflammatory therapies would be beneficial in the treatment of T2DM and the prevention of CVD, yet the success of antiinflammatory therapies in the treatment of cardiometabolic disorders has been limited (6).

The ability of VAT to stimulate this cycle of inflammation and insulin resistance is underscored by the syndrome of “normal-weight” obesity: patients with normal BMIs but increased waist circumference and disproportionately increased VAT (7). Despite having a normal BMI these patients are prone to insulin resistance and T2DM because of excess VAT. These observations have led to interest in the direct adverse effects of VAT on neighboring organs. For example, epicardial adipose tissue (EAT) is the visceral fat depot of the heart (8). Modest amounts of EAT are present in normal-weight humans; in overweight humans the amount of EAT increases proportionately with obesity and VAT (9). EAT is contained within the visceral pericardium and supports the epicardial coronary arteries and great cardiac veins (8). Early studies by Mazurek et al. demonstrated that EAT surrounding atherosclerotic coronary arteries had increased expression of inflammatory genes and dense inflammatory cell infiltrates compared with subcutaneous adipose tissue (SAT) (10). This observation suggested that inflammation of EAT may contribute to or exacerbate atherosclerosis in the underlying coronary artery. Many other groups have replicated these results, finding increased inflammatory gene expression in the EAT of patients with coronary artery disease (CAD) (11–14). However, there are some limitations to these prior studies. First, control patients without CAD were not routinely included. This is important because inflammation in VAT is typically greater than SAT even in normal conditions (15). Second, it impossible to discern the temporal relationship between the development of CAD and inflammation in adjacent EAT. Therefore, to address some of these limitations, we chose to directly compare gene expression in the EAT of patients with and without CAD.

We hypothesized that inflammatory gene expression would be greater in the EAT of patients with CAD. Contrary to our hypothesis, we found a greater number of genes differentially expressed in the EAT of patients *without CAD*. In particular, we found increased expression of all 3 members of the NR4A subfamily of orphan nuclear hormone receptors (*NR4A1*, *NR4A2*, *NR4A3*) in the EAT of those without CAD. Members of this subfamily have been found to suppress inflammatory gene expression (16, 17). For example, whole-body and macrophage-specific deletion of *Nr4a1* in mice results in a worsening of atherosclerosis (17, 18). Therefore, we conclude that the development of CAD may be associated with *decreased antiinflammatory* gene expression in EAT. Promotion of antiinflammatory effects via pharmacological activation of the orphan nuclear hormone receptors may be advantageous in the treatment of CAD and other cardiometabolic disorders and will be the focus of future studies.

## Results

### Clinical characteristics.

The clinical characteristics of subjects are shown in Table 1. There was no difference in age, gender, BMI, comorbid conditions, or pertinent medication use. The mean age of controls was 67.1 ± 9.9 years and cases 65.3 ± 13.0 years (*P* = 0.70). There was a trend toward more female patients in the control group (46% vs. 23% for cases, *P* = 0.41). The mean Gensini score was 0.7 ± 1.3 in controls and 53.7 ± 27.2 in cases (*P* < 0.001). Therefore, with the exception of the presence of CAD, our study groups were well matched.

### EAT contains smaller adipocytes and is more vascularized.

**Figure 1:**
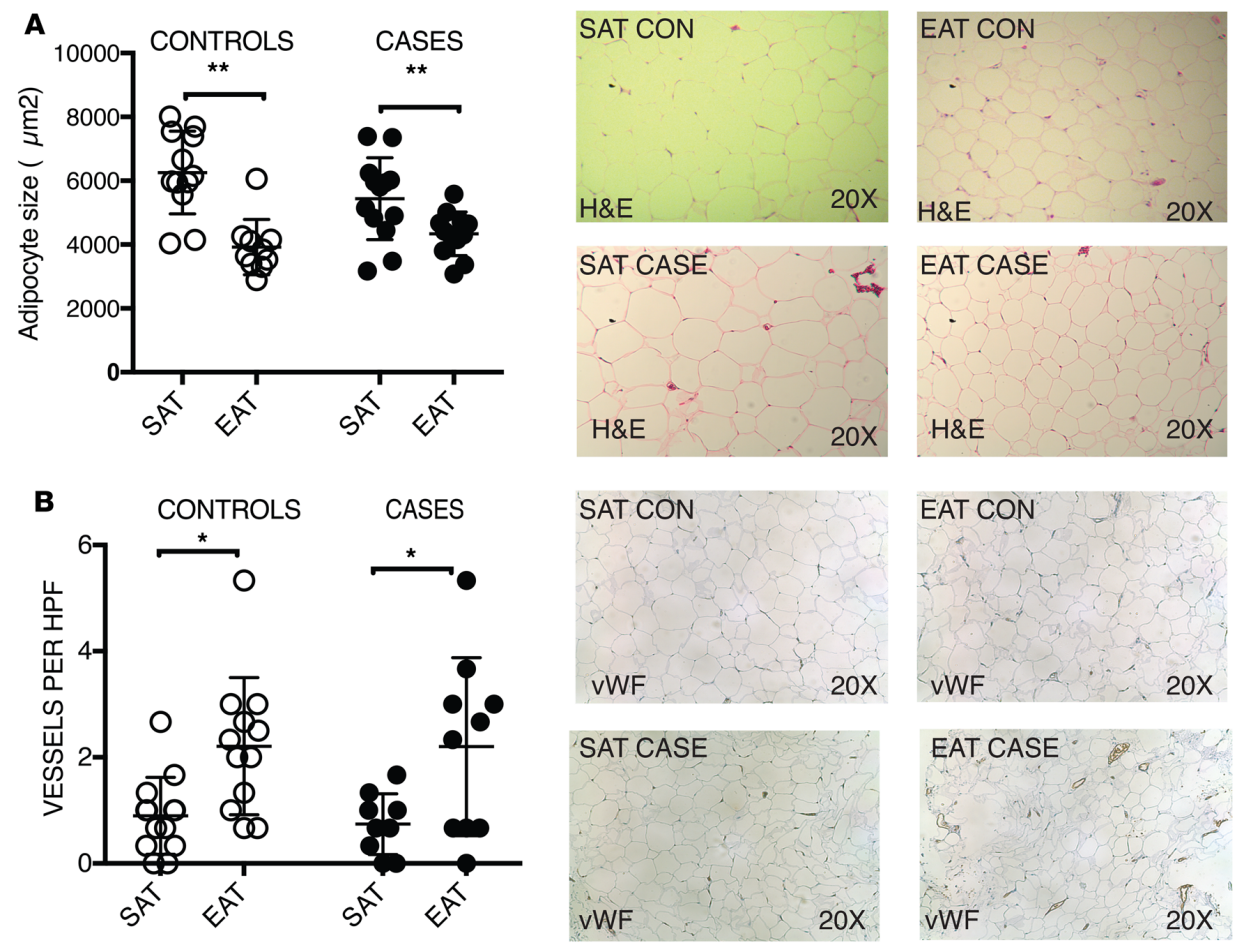
EAT contains smaller adipocytes and greater number of blood vessels. (**A**) Adipocyte size was determined using Adiposoft software on H&E-stained sections of SAT and EAT in both cases and controls (top right). The mean adipocyte size in SAT was larger than those in EAT (top left). This was true for both cases and controls. (**P < 0.01 SAT vs. EAT, 2-tailed Student’s *t* test; *n* = 13 per group. In each column, individual subjects are plotted, and error bars show the mean and standard deviation per group). (**B**) Sections of SAT and EAT were stained with vWF, and the number of blood vessels per high-power field (HPF) was quantified (bottom right). EAT had more blood vessels per field than SAT. (*P < 0.05 EAT vs. SAT, 2-tailed Student’s *t* test; *n* = 13 per group. In each column, individual subjects are plotted, and error bars show the mean and standard deviation per group).

We first compared the histological characteristics of SAT and EAT. Both SAT and EAT contained unilocular white adipocytes (Figure 1); no brown adipocytes were visualized. Control SAT adipocytes were significantly larger than those in EAT (5213 ± 1024 μm^2^ vs. 3904 ± 1241 μm^2^, *P* < 0.01) (Figure 1). This relationship was also seen in cases (5136 ± 1058 μm^2^ vs. 3920 ± 612 μm^2^, *P* < 0.01). There was no difference between cases and controls in depot-specific adipocyte size. We then used an endothelial-specific antibody (von Willebrand factor) to compare the number of blood vessels in SAT and EAT (Figure 1). EAT had a greater number of blood vessels than SAT; this was true for cases (2.2 ± 1.67 vs. 0.66 ± 0.58 per HPF, *P* < 0.01) and controls (2.2 ± 1.29 vs. 0.89 ± 0.72 per HPF, *P* < 0.01). There was no difference between cases and controls in depot-specific blood vessel number.

### Microarray analysis demonstrates predominantly depot-specific differences in gene expression.

**Figure 2:**
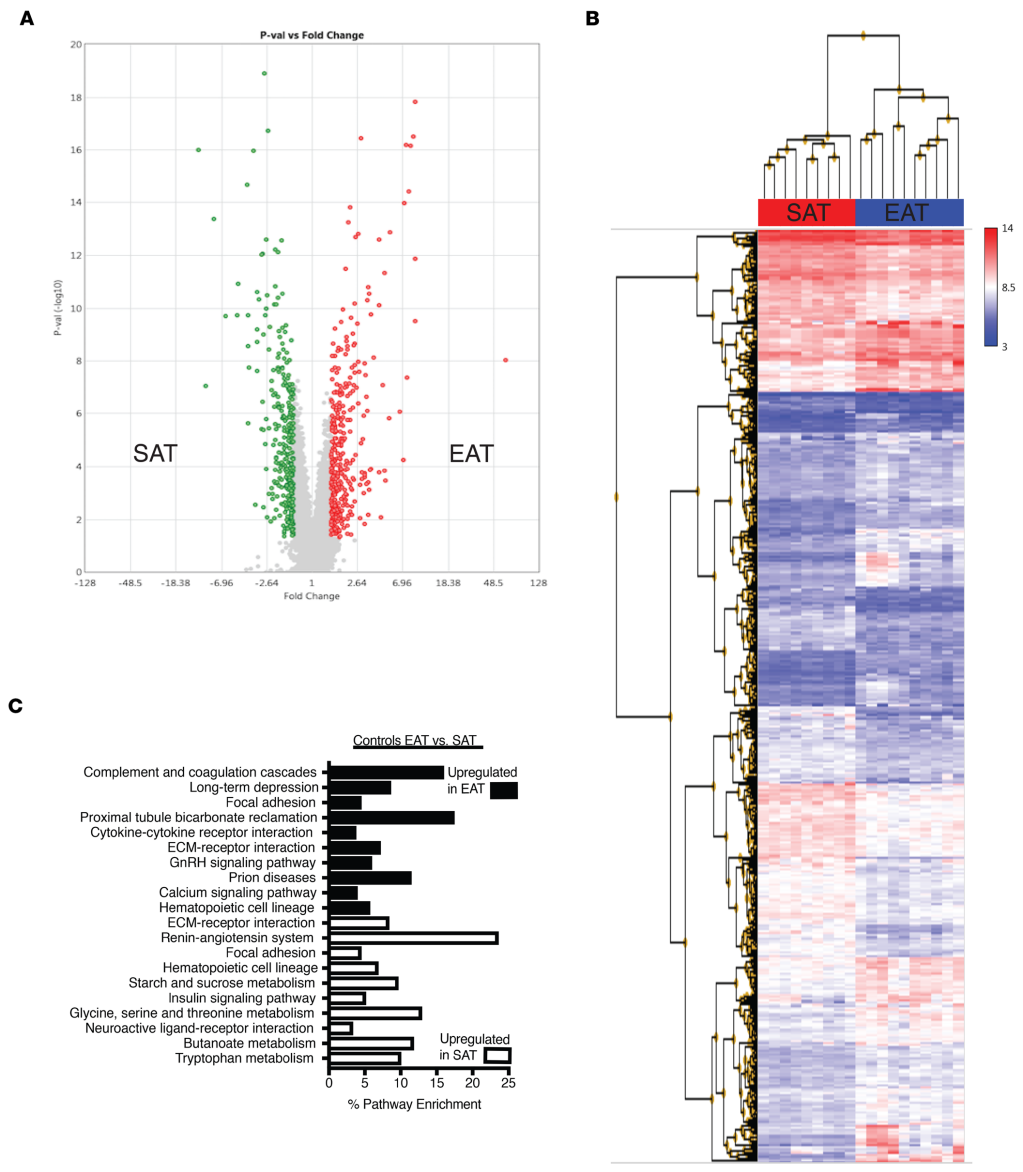
Comparison of gene expression between SAT and EAT in controls. (**A**) Volcano plot of 693 probes that were differentially expressed at a level of 1.5-fold change (FC) (ANOVA *P* < 0.05). In EAT (shown in red), 383 were upregulated, and 310 were upregulated in SAT (shown in green). (**B**) Hierarchical clustering of differentially expressed genes in control SAT and EAT. (**C**) KEGG pathway analysis of differentially expressed genes in SAT and EAT.

We then compared gene expression in SAT and EAT in control patients. Despite the absence of significant CAD, there were large differences in gene expression between SAT and EAT (Figure 2 and Supplemental Table 1; supplemental material available online with this article; https://doi.org/10.1172/jci.insight.124859DS1). In total, 693 genes were differentially expressed (FC > 1.5; *P* < 0.05). Expression of 383 genes was increased in EAT and 310 were increased in SAT. We then performed gene set enrichment analysis (GSEA) of differentially expressed genes in both depots. SAT was enriched for expression of genes encoding proteins related to the renin-angiotensin system, neuroactive receptor-ligand interactions, and many biochemical pathways (insulin signaling, starch, sucrose, and butanoate metabolism) (Figure 2C). In contrast, EAT was enriched for expression of genes encoding inflammatory pathways, such as complement and coagulation cascades, focal adhesion, and cytokine-receptor interaction (Figure 2C).

**Figure 3:**
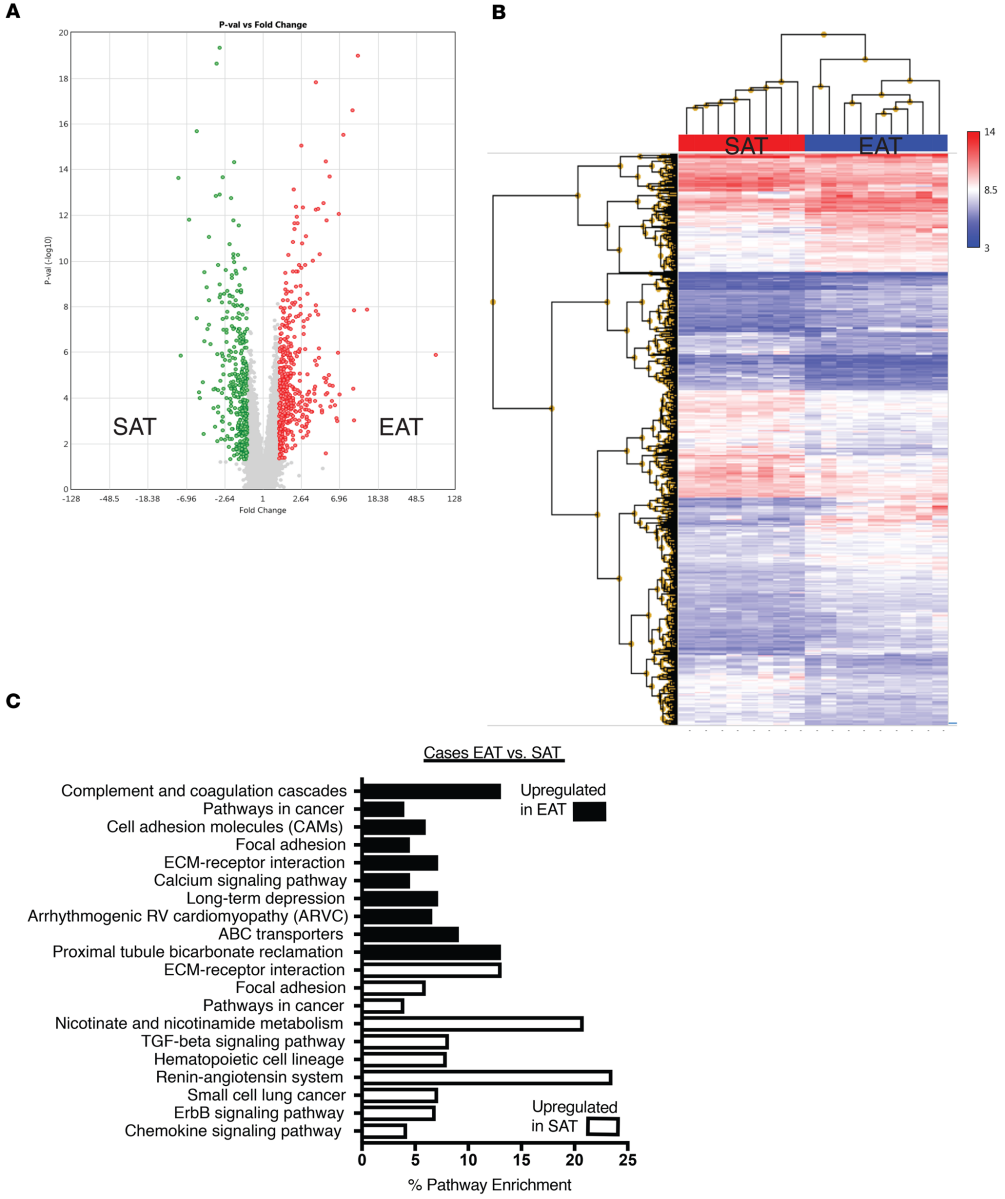
Comparison of gene expression between SAT and EAT in cases. (**A**) Volcano plot of 805 genes that were differentially expressed at a level of 1.5-fold change (ANOVA *P* < 0.05). In EAT (red) 427 were upregulated, and 378 were upregulated in SAT (green). (**B**) Hierarchical clustering of differentially expressed genes in SAT and EAT of cases. (**C**) KEGG pathway analysis of differentially expressed genes in SAT and EAT of cases.

In patients with CAD 805 genes were differentially expressed between SAT and EAT (Figure 3 and Supplemental Table 1). Expression of 427 was increased in EAT and 328 in SAT. GSEA demonstrated that SAT was enriched for expression of genes encoding proteins implicated in the renin-angiotensin system, nicotinate and nicotinamide metabolism, and TGF-β signaling pathways (Figure 3C). Similar to control patients, EAT was enriched for expression of genes encoding proteins belonging to complement and coagulation cascades, cell adhesion molecules, focal adhesion and calcium signaling pathways (Figure 3C).

**Figure 4:**
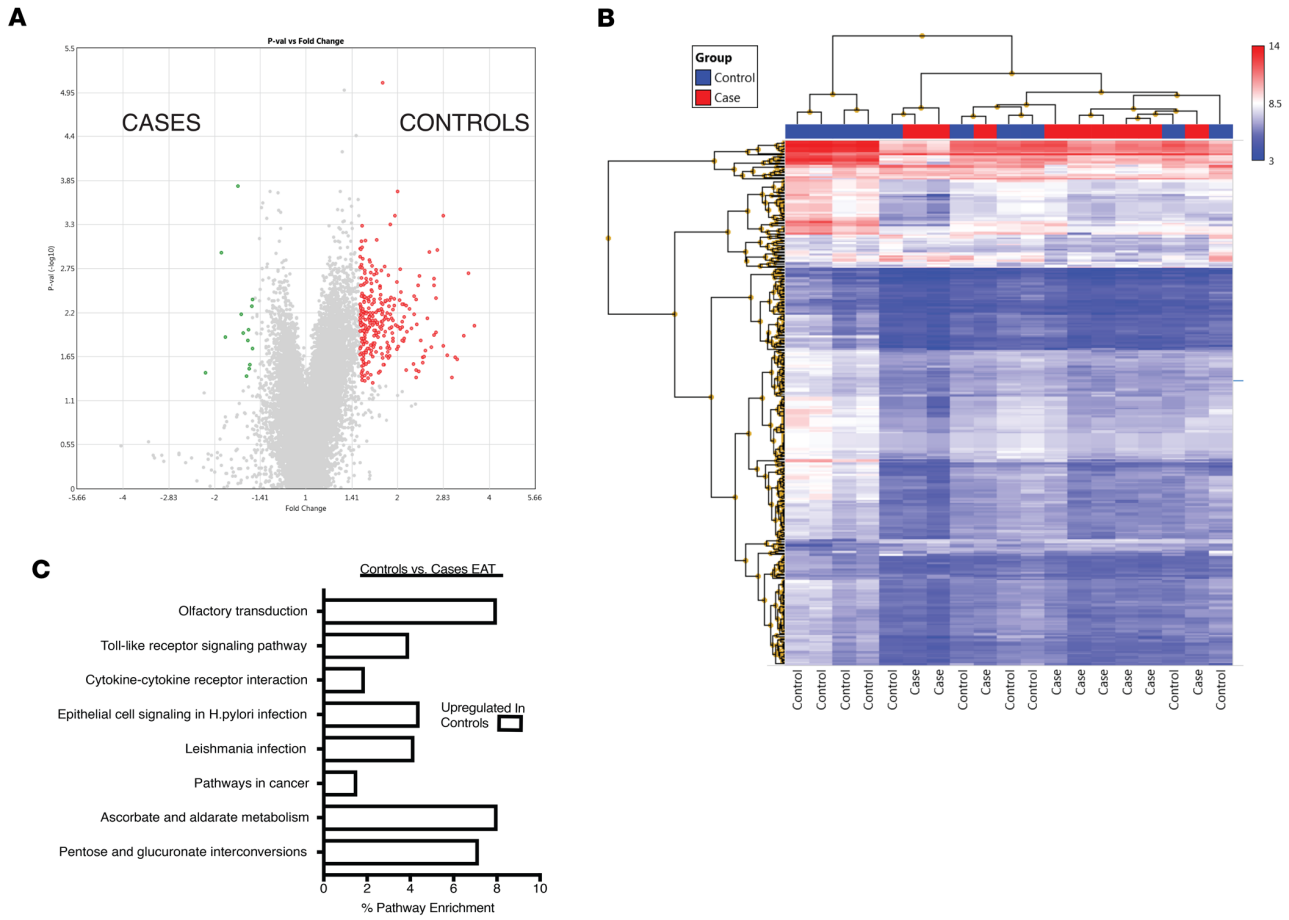
Comparison of gene expression in EAT between cases and controls. (**A**) Volcano plot of 326 genes that were differentially expressed at a level of 1.5-fold change (ANOVA *P* < 0.05). Three hundred twelve were upregulated in the EAT of control patients (red) and 14 in the EAT of cases (green). (**B**) Hierarchical clustering of differentially expressed genes in EAT of cases and controls. (**C**) KEGG pathway analysis of differentially expressed genes in EAT of controls. No pathways were significantly enriched in the EAT of cases.

We then directly compared the gene expression profile of EAT in cases and controls (Figure 4). Three hundred twenty-six genes were differentially regulated (1.5-fold change, *P* < 0.01). Expression of 312 genes was increased in controls and 14 in cases (Figure 4A, Table 1, and Supplemental Table 1). Because of the small number of genes differentially regulated in the EAT of cases, no Kyoto Encyclopedia of Genes and Genomes (KEGG) pathway was significantly enriched in this depot (Figure 4C). However, several pathways were increased in the EAT of controls, including olfactory transduction, pentose and glucuronate interconversion, and Toll-like receptor transduction (Figure 4C).

**Figure 5:**
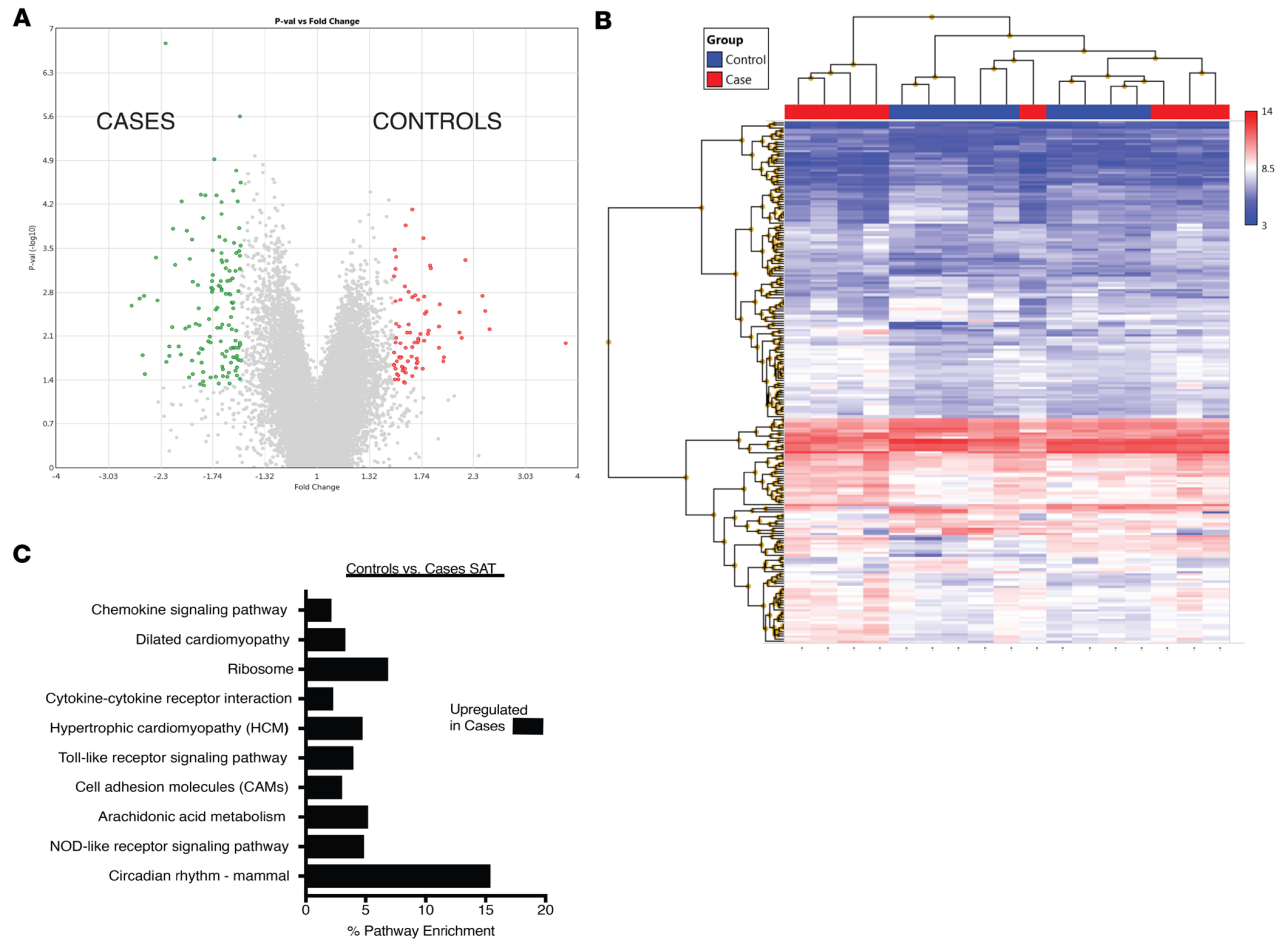
Comparison of gene expression in SAT between cases and controls. (**A**) Volcano plot of 218 genes that were differentially expressed at a level of 1.5-fold change (ANOVA *P* < 0.05). Eighty-two were upregulated in the SAT of control patients (red) and 136 in the SAT of cases (green). (**B**) Hierarchical clustering of differentially expressed genes in SAT of cases and controls. (**C**) KEGG pathway analysis of differentially expressed genes in SAT of cases. No pathways were significantly enriched in the SAT of controls.

We then compared gene expression in SAT between cases and controls (Figure 5). Expression of 218 genes was significantly different; expression of 82 was increased in the SAT of controls and 136 in the SAT of cases (Figure 5A). No pathways were significantly enriched in the SAT of controls; several pathways were increased in the SAT of cases, including those encoding proteins implicated in circadian rhythms, chemokine signaling, and NOD receptor signaling (Figure 5C).

Finally, an advantage of the Human Gene 1.0 array is that it contains probes for MIRs. Therefore, we used this feature to compare MIR expression in SAT and EAT in cases and controls (Table 2 and Table 3). Expression of MIRs 126 and 1247 was significantly upregulated in the EAT of controls and cases compared with respective SAT. MIR24-2 expression was increased in the SAT of cases and controls in comparison with EAT.

Therefore, contrary to our expectations, microarray analysis did not demonstrate greater inflammatory gene expression in the EAT of cases in comparison to controls. Rather, depot-specific differences predominated, with SAT of both cases and controls enriched in certain pathways (renin-angiotensin system, focal adhesion) and EAT of both cases and controls enriched in others (complement and coagulation, calcium signaling) (Figure 2 and Figure 3).

### Quantitative reverse transcription PCR and immunohistochemistry show that there is no difference in inflammation between EAT of cases and controls.

**Figure 6:**
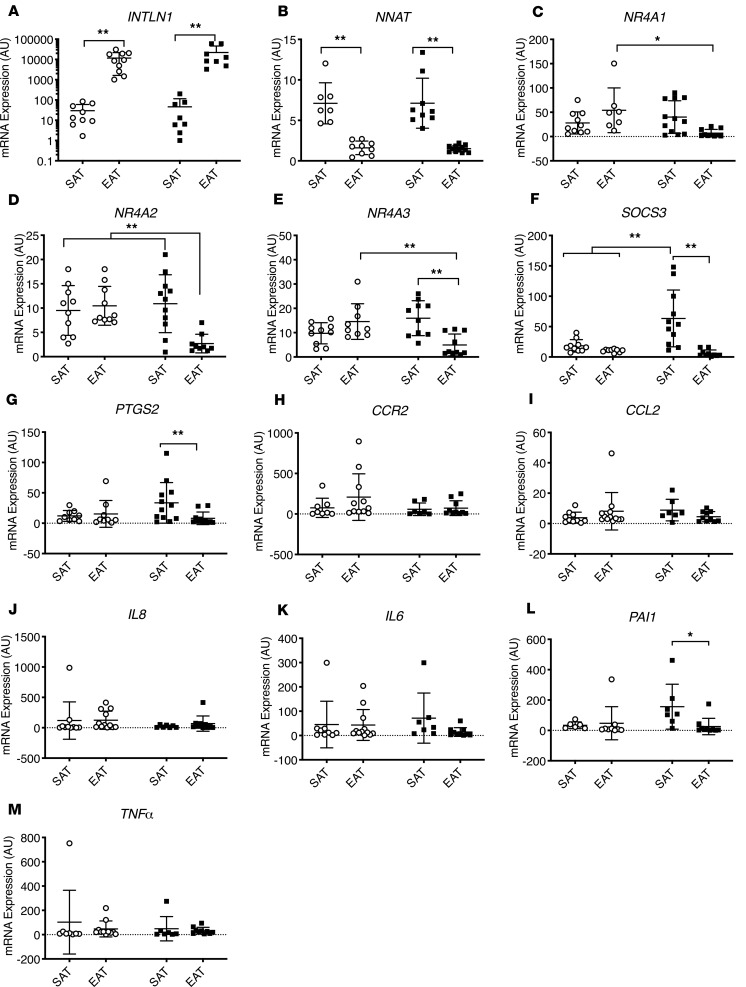
qRT-PCR verifies downregulation of *NR4A1*, *NR4A2*, and *NR4A3* in EAT of cases. (**A**) *INTLN1* expression was greater in EAT than SAT in both cases and controls. (**B**) *NNAT* expression was greater in SAT than EAT in both cases and controls. (**C**) *NR4A1* expression was significantly decreased in EAT of cases compared with controls. (**D**) *NR4A2* expression was reduced in EAT of cases in comparison with all other depots. (**E**) *NR4A3* expression was reduced in EAT of cases in comparison with SAT of cases and EAT of controls. (**F**) *SOCS3* expression was increased in the SAT of cases compared with other groups. (**G**) *PTGS2* expression was increased in the SAT of cases compared with EAT. (**H**) *CCR2* expression was not different between groups. (**I**) *CCL2* expression was not different between groups. (**J**) *IL8* expression was not different between groups. (**K**) *IL6* expression was not different between groups. (**L**) *PAI1* expression was not different between groups. (**M**) *TNFA* expression was not different between groups (*n* = 11–12 per group; **P < 0.01, and *P < 0.05 for indicated comparisons using 2-way ANOVA and Tukey’s multiple-comparisons test). In each column, individual subjects are plotted, and error bars show the mean and standard deviation per group. White circles represent controls; black squares represent cases.

We performed quantitative reverse transcription (qRT-PCR) to verify the expression patterns that we observed in the microarray experiments. First, we showed that our samples displayed a depot-specific identity. EAT of both cases and controls had a higher expression of the visceral fat marker omentin-1 (*INTLN1*) (Figure 6A). In contrast, the SAT of cases and controls expressed higher levels of the subcutaneous fat marker neuronatin (*NNAT*) (Figure 6B). Expression of common inflammatory genes (*CCR2*, *CCL2*, *IL8*, *IL6*, *PAI1*, and *TNFA*) was not significantly different between groups (Figure 6). Interestingly, expression of 3 members of the orphan nuclear hormone receptor family was increased in the EAT of control patients in comparison with cases (Figure 6, C–E).

**Figure 7:**
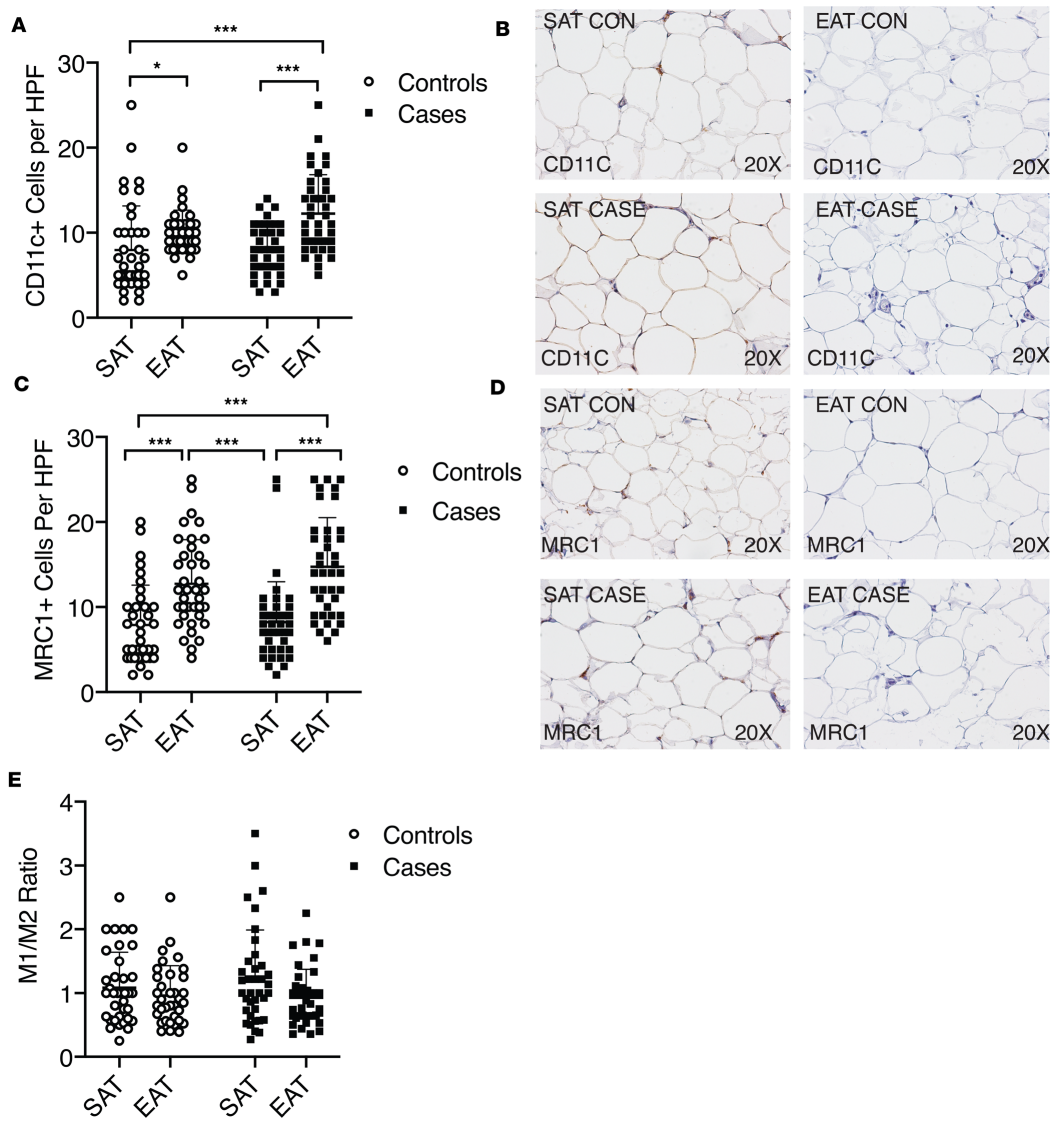
Staining of EAT for classical and alternative macrophage markers shows no difference between cases and controls. (**A**) Staining of EAT for the classical macrophage marker CD11c showed no difference between cases and controls. EAT of both cases and controls had more CD11c^+^ cells than SAT. *P < 0.05; ***P < 0.001 vs. EAT for indicated comparisons using Kruskal-Wallis test with Dunn’s multiple-comparisons test. In each column, individual subjects are plotted, and error bars show the mean and standard deviation per group. (**B**) Representative CD11c staining in cases and controls. (**C**) Staining of EAT for the alternative macrophage marker MRC1 showed no difference between cases and controls. EAT of both cases and controls had a greater number of MRC1^+^ cells than SAT. ***P < 0.001 vs. EAT for indicated comparisons using Kruskal-Wallis test with Dunn’s multiple-comparisons test. In each column, individual subjects are plotted, and error bars show the mean and standard deviation per group. (**D**) Representative MRC1 staining in cases and controls. (**E**) The average values or CD11c and MRC1^+^ cells per subject and tissue were divided to create a ratio. There was no difference in the ratio per tissue or group using Kruskal-Wallis test.

We then performed immunohistochemistry for markers of classical (M1) and alternative (M2) macrophage activation. In both cases and controls, EAT had a significantly greater number of classical (CD11c^+^) and alternative (mannose receptor C-type 1–positive [MRC1^+^]) macrophages than SAT (Figure 7). There was no difference in the number of positive-stained cells between the EAT of cases and controls (Figure 7). Furthermore, we did not observe a difference in the ratio of M1/M2 cells per depot or on a disease-specific basis (Figure 7E).

## Discussion

There has been much recent interest in the potential role of EAT as a direct link between obesity and CAD (19, 20). Initial studies showed that EAT had greater mRNA and protein expression of inflammatory genes and clusters of inflammatory cells (10, 21). Subsequent analyses have demonstrated that EAT tends to have a more inflammatory gene expression profile than SAT, and this is true even in the absence of CAD (12–14). The majority of the data suggest that EAT is very similar to abdominal VAT in that it tends to be more immunologically active than SAT even in normal conditions. There may be an additional increase in inflammation in the setting of atherosclerosis, but it is the adipose organ itself, not the disease state, that dictates the majority of differences in gene expression (13, 14). This concept was originally proposed by Chatterjee et al. in 2009 (22).

Our study confirms many of these findings and makes several potentially novel observations. First, we confirmed that type of adipose tissue, rather than the disease condition, is responsible for most of the differences in gene expression. Six hundred ninety-three genes were differentially expressed between SAT and EAT in controls and 805 in cases. In particular, there is a hallmark panel of genes whose expression is consistently increased in EAT (*INTLN1*, *SYT4*, *CFB*, *ESR*, *INMT*, *PRG4*, and *ALOX15*) compared with SAT irrespective of the presence or absence of CAD (13). In contrast, when directly comparing the EAT of cases and controls, there are fewer differences in gene expression (326 probes different). However, among these differences, we found that expression of 3 members of the NR4A subfamily of orphan nuclear hormone receptors was increased in the EAT of patients *without CAD*. This has not been previously reported to our knowledge and is very interesting considering that members of this family have been shown to be protective in mouse models of atherosclerosis (17, 18).

The NR4A subfamily of orphan nuclear hormone receptors belong to the same class of transcription factors as peroxisome proliferator–activated receptor γ. Their protein structure is similar to other members in this class, having separate DNA- and ligand-binding domains. They are called “orphan” receptors because their endogenous ligands have yet to be discovered. NR4A family members are expressed in a broad array of tissues and have been shown to protect against the initiation and progression of atherosclerosis (23, 24). Recent studies have highlighted *NR4A1* as a key transcriptional regulator of lipid homeostasis, vascular remodeling, and inflammation (25). *NR4A1* has at least 3 important antiinflammatory effects relevant to CAD. First, NR4A1 activity is required for the differentiation of Ly6c^–^ monocytes that patrol the endothelium and maintain blood vessel integrity. Nr4a1-null mice lack this antiinflammatory monocyte population and display an exaggerated inflammatory response (16, 17). Second, within Ly6c^+^ monocytes, Nr4a1 feeds back to inhibit inflammasome activity and transactivation of NF-κB by IL-2. Third, Nr4a1 suppresses induction of macrophage chemotactic protein-1 expression in response to lipopolysaccharide (22). Although we do not know the specific cell type responsible for NR4A1 expression in EAT, we hypothesize that it is macrophages. Hirata et al. reported that the ratio of M2 (alternatively activated) to M1 (classically activated) macrophages is greater in the EAT of patients without CAD (21). The inflammatory phase of myocardial infarction (MI) is characterized by influx of Ly6c^hi^CCR2^hi^ monocytes (27). The transition to the reparative phase of MI is dependent upon NR4A1 downregulation of inflammatory cytokine production and transition to Ly6c^lo^CCR2^lo^ macrophages. Deletion of NR4A in this context leads to excessive inflammation and maladaptive post-MI remodeling (27). Therefore, we hypothesize that within the EAT of patients without CAD, there exists a greater population of antiinflammatory macrophages. Determining whether this is true will require further research.

We showed that epicardial adipocytes are smaller than subcutaneous adipocytes, which is consistent with their visceral lineage. This confirms work by prior authors (28). We did not, however, see a difference in the size of epicardial adipocytes between cases and controls; this is in contrast to the work of Vianello et al., who demonstrated that epicardial adipocyte size was greater in patients with CAD than in those without (29). We hypothesize that this could be due to differences in the patient population. Vianello et al. included only male patients in their study, and the average BMI of patients in their study was lower (29).

A potentially novel finding in our study is that EAT appears to have a greater number of blood vessels than SAT. This has not been previously shown. This was unexpected because visceral adipose depots have previously been demonstrated to have a lower blood vessel density than SAT (30). However, it may be that EAT is different from abdominal VAT in this regard. Furthermore, EAT is known to have a blood supply from the underlying coronary arteries; therefore it follows that there may be a higher vascularity in this adipose depot, which is in close proximity to the heart (8).

Interestingly, we found that the expression level of MIR126 was higher in the EAT of both cases and controls compared with SAT. MIR126 is one of the most abundant MIRs in endothelial cells (31). MIR126 promotes regeneration of endothelial cells and regulates endothelial cell turnover (32). The increased expression of this MIR in EAT is consistent with the increased number of endothelial cells in this depot.

Our study has several limitations. First, we have a modest number of patients. Second, we could not obtain samples of visceral fat to compare the 3 depots. Third, samples of EAT were obtained proximal to the right coronary artery only, because this is the easiest area for the surgeon to take the biopsy from. These limitations aside, we feel that we have made some potentially novel observations regarding the characteristics of EAT that add to the present literature.

### Conclusions.

We conclude that the adipose depot, rather that disease status, is the predominant determinant of gene expression in EAT. EAT has greater inflammatory gene expression than SAT even in the absence of coronary disease; this is consistent with its visceral origin. Regarding disease-specific gene expression, patients without coronary disease had higher expression of all 3 members of the orphan nuclear hormone receptor family in EAT. This is consistent with mouse studies demonstrating a protective and antiinflammatory effect of these nuclear receptors in atherosclerosis. We hypothesize that these receptors may play an important role in limiting adipose tissue inflammation in cardiometabolic disorders.

## Methods

### Subject recruitment.

Adult patients referred for elective cardiac surgery were screened for enrollment and informed consent was provided. This study was approved by the UMass IRB (docket H-14436). Patients with active infection or cancer were excluded. All patients had preoperative coronary angiography. Control patients were referred for elective valve surgery and had no significant CAD (any single lesion >50%) on preoperative coronary angiograms. Only 3 controls had single lesions of 30%; otherwise, all had normal coronary arteries or minor luminal irregularities. Cases were patients referred for coronary artery bypass surgery because of significant CAD. The severity of CAD was determined by single-blinded review of coronary angiograms and calculation of the Gensini score as previously described (33). The Gensini score is a continuous variable that increases with the severity of coronary atherosclerosis (e.g., a score of 0 = none, and 100 = severe).

### Sample collection and preparation.

During surgery a sample of EAT (0.5 g) adjacent to the right coronary artery was obtained, a section was taken and stored in 10% formalin, and the remainder was immediately snap-frozen in liquid nitrogen (–80°C). Next, a sample of SAT (0.5 mg) from thoracic subcutaneous fat was obtained, a section was taken and stored in 10% formalin, and the remainder was immediately snap-frozen in liquid nitrogen (–80°C).

### Microarray analysis.

RNA was prepared from aliquots of frozen fat samples using the QIAGEN mini lipid RNA extraction kit. RNA quality was analyzed on an Agilent Bioanalyzer. Samples with an RNA integrity number below 7.5 were excluded. Samples that passed quality control analysis were sent to the UMass Genomics Core for cRNA preparation and hybridization to Affymetrix Human Gene 1.0 Arrays. Expression analysis was performed using Affymetrix Transcriptome Analysis Software. The raw data were deposited into the National Center for Biotechnology Information’s Gene Expression Omnibus database with accession number GSE120774 (http://www.ncbi.nlm.nih.gov/geo).

### qRT-PCR.

qRT-PCR was performed using a cDNA synthesis kit, SYBR Green Master Mix, and a CFX 96 thermocycler (all from Bio-Rad) as previously described (34). Primer sequences were obtained from PrimerBank (http://pga.mgh.harvard.edu/primerbank) and were as follows: *CCR2* forward CCACATCTCGTTCTCGGTTTATC, *CCR2* reverse CAGGGAGCACCGTAATCATAATC, *CCL2* forward CAGCCAGATGCAATCAATGCC, *CCL2* reverse TGGAATCCTGAACCCACTTCT, *GAPDH* forward GGAGCGAGATCCCTCCAAAAT, *GAPDH* reverse GGCTGTTGTCATACTTCTCATGG*, IL8* forward TTTTGCCAAGGAGTGCTAAAGA, *IL8* reverse AACCCTCTGCACCCAGTTTTC, *IL6* forward ACTCACCTCTTCAGAACGAATTG, *IL6* reverse CCATCTTTGGAAGGTTCAGGTTG, *ITLN1* forward ACGTGCCCAATAAGTCCCC, *ITLN1* reverse CCGTTGTCAGTCCAACACTTTC, *NNAT* forward ACTGGGTAGGATTCGCTTTTCG, *NNAT* reverse ACACCTCACTTCTCGCAATGG, *NR4A1* forward ATGCCCTGTATCCAAGCCC, *NR4A1* reverse GTGTAGCCGTCCATGAAGGT, *NR4A2* forward GTTCAGGCGCAGTATGGGTC, *NR4A2* reverse CTCCCGAAGAGTGGTAACTGT, *NR4A3* forward TGCGTCCAAGCCCAATATAGC, *NR4A3* reverse GGTGTATTCCGAGCTGTATGTCT, *PAI1* forward ACCGCAACGTGGTTTTCTCA, *PAI1* reverse TTGAATCCCATAGCTGCTTGAAT, *PTGS2* forward CTGGCGCTCAGCCATACAG, *PTGS2* reverse CGCACTTATACTGGTCAAATCCC, *SOCS3* forward CCTGCGCCTCAAGACCTT, *SOCS3* reverse GTCACTGCGCTCCAGTAGAA, *TNFA* forward CCTCTCTCTAATCAGCCCTCTG, and *TNFA* reverse GAGGACCTGGGAGTAGATGAG. Expression relative to *GAPDH* was determined using the ΔΔCt method of Livak and Schmittgen (35).

### Histology and immunohistochemistry.

Formalin-fixed sections were embedded in paraffin and sectioned by the UMass Diabetes and Endocrinology Research Center (DERC) Morphology Core. Adipocyte size was determined on H&E-stained sections using the program Adiposoft as previously described (36). To quantify blood vessels, unstained sections were incubated with rabbit anti–human von Willebrand factor (1:300 dilution) (Abcam ab6994) and then a goat antirabbit secondary antibody conjugated to horseradish peroxidase (Pierce 31460). Negative controls using secondary antibody alone were also included. To quantify M1 or M2 macrophages, cryosections were stained with rabbit anti–human CD11c (1:1000 dilution) (abcam ab52632) and rabbit anti–human MRC1 (1:1000 dilution) (abcam ab64693) antibodies and then a goat antirabbit secondary antibody conjugated to horseradish peroxidase. Three representative fields at original magnification ×20 of SAT and EAT of each patient were used to quantify CD11c^+^ and MRC1^+^ cells.

### Statistics.

Clinical characteristics were compared with the 2-tailed Student’s *t* test for continuous variables or the χ^2^ test for dichotomous variables. A *P* value less than 0.05 was considered significant. Adipocyte size and endothelial cell numbers were compared with 2-tailed Student’s *t* test. For the qRT-PCR experiment, expression values relative to *GAPDH* were compared using 2-way ANOVA and Tukey’s multiple-comparisons test. The Affymetrix Transcriptome Analysis Console was used to analyze microarrays using 1-way ANOVA and multiple-comparisons testing.

### Study approval.

This study was reviewed and approved by the UMass Medical Institutional Review Board (docket H-14436). All studies were conducted according to the principles expressed in the Declaration of Helsinki. Signed informed consent documents were obtained from each participant included in the study.

## Author contributions

TPF, SKCT, and MPC conceived and designed the experiments. TPF, NL, KVT, SN, and MK performed the experiments. TPF, NL, KVT, SN, and MK analyzed the data. TPF was responsible for consenting patients, and TPF and SKCT obtained samples. TPF and MPC wrote the paper.

## Supplementary Material

Supplemental Table 1

## Figures and Tables

**Table 3:**
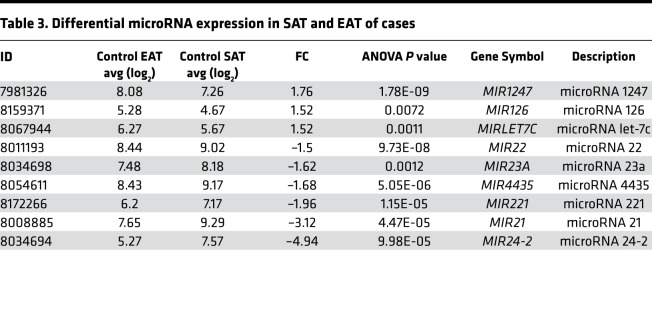
Differential microRNA expression in SAT and EAT of cases

**Table 2:**
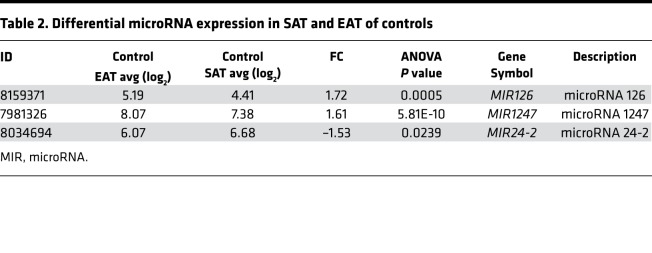
Differential microRNA expression in SAT and EAT of controls

**Table 1:**
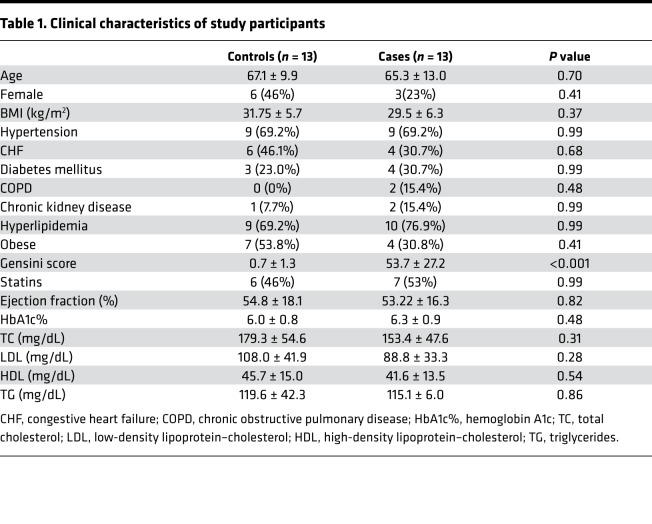
Clinical characteristics of study participants

## Version 1 09/12/2019

In-Press Preview

## Version 2 10/17/2019

Electronic publication
